# Ultra-early neurological pupil index trajectories and clinical outcomes in acute brain injury

**DOI:** 10.3389/fneur.2026.1741572

**Published:** 2026-06-12

**Authors:** Yong Soo Kim, Dong-Wan Kang, Hyung Seok Guk, Museong Kim, Heewon Jeong, Huimahn Alex Choi, Sung-Min Cho, Moon-Ku Han, Hee Eun Kim, Dong Keon Lee, Han-Gil Jeong

**Affiliations:** 1 Division of Intensive Care Medicine, Departments of Neurosurgery and Neurology, Seoul National University Bundang Hospital, Seoul National University College of Medicine, Seongnam-si, Gyeonggi-do, Republic of Korea; 2 Department of Neurosurgery, Chungnam National University Hospital, Daejeon, Republic of Korea; 3 Department of Neurosurgery, McGovern Medical School, The NABI Institute, University of Texas Health Science Center at Houston, Houston, TX, United States; 4 Division of Neurosciences Critical Care, Departments of Neurology, Surgery, Anaesthesiology and Critical Care Medicine and Neurosurgery, Johns Hopkins University School of Medicine, Baltimore, MD, United States; 5 Department of Emergency Medicine, Seoul National University Bundang Hospital, Seoul National University College of Medicine, Seongnam, Gyeonggi-do, Republic of Korea

**Keywords:** acute brain injury, automated pupillometry, hyperacute phase, neurological pupil index, trajectory

## Abstract

**Background and purpose:**

To investigate the association between ultra-early quantitative pupillometry indices measured within the first 24 h of emergency department (ED) arrival and clinical outcomes in patients with acute brain injury.

**Methods:**

This retrospective analysis of a prospectively collected database assessed patients admitted to the ICU of a tertiary hospital for acute brain injury (aneurysmal subarachnoid hemorrhage, traumatic brain injury, intracerebral hemorrhage, and ischemic stroke) between September 2022 and July 2024. Automated pupillometry was performed at 0, 1, 3, 6, and 24 h after ED arrival. Neurological pupil index (NPi) trajectories were categorized into four clusters (*Consistently high*, *Worsened*, *Recovered*, and *Consistently low*) using k-means longitudinal clustering. The primary outcome was ICU mortality; secondary outcomes were 6-month mortality and poor functional outcome (modified Rankin Scale 4–6). Associations of time-specific pupillometry indices and their longitudinal trajectories with outcomes were investigated.

**Results:**

Among 168 patients [age 69.6 ± 15.2 years; arrival GCS 7 (5–9); onset-to-arrival time 1.6 (0.9–5.2) h], ICU mortality was 29.8%. The *Worsened* and *Consistently low* NPi trajectory groups exhibited higher ICU mortality compared to the *Consistently high* group, while the *Recovered* group showed no significant difference. Incorporating NPi trajectories improved predictive accuracy for ICU mortality (AUROC, 0.89 vs. 0.80, *p* < 0.01), 6-month mortality (0.83 vs. 0.76, *p* = 0.01), and poor functional outcomes (0.87 vs. 0.82, *p* = 0.06) compared to a model using clinical variables alone.

**Conclusion:**

Ultra-early changes in pupillometry indices during the hyperacute phase enhance risk stratification and improve outcome prediction in acute brain injury.

## Introduction

Acute brain injuries, including traumatic brain injury (TBI), intracerebral hemorrhage (ICH), aneurysmal subarachnoid hemorrhage (SAH), and ischemic stroke, are major causes of morbidity and mortality (1). The neurological status in acute brain injury is highly dynamic, particularly within the first 24 h of admission (2–4). Approximately 10% of patients with acute brain injury exhibit neurological deterioration during their emergency department (ED) stay, and this deterioration is closely associated with increased mortality and poor functional outcomes (5–7). Since neurological deterioration often reflects intracranial pressure (ICP) elevation or exacerbating brain injury, detection and monitoring of neurological changes in the critical period is essential for establishing an initial treatment strategy (8, 9). However, due to initial symptoms such as impaired consciousness or inability to follow commands, recognizing these changes in clinical settings is often delayed or challenging.

Assessment of pupillary light reflexes is a key component in monitoring the progression of brain injury. However, traditional penlight examinations are prone to high inter-observer variability and low reliability, limiting their clinical utility (10). Automated pupillometry, which quantifies pupillary light reflexes, has emerged as a preferred method due to its consistency and minimal interference by sedatives such as propofol and remifentanil (11, 12). Among automated pupillometry-derived parameters, the neurological pupil index (NPi) is a composite score ranging from 0 to 5 that summarizes the pupillary light reflex, with values below 3 considered abnormal. Because NPi provides a standardized, quantitative measure of pupillary reactivity, it may support objective neurological assessment when the conventional neurological examination is limited by impaired consciousness or sedation. Additionally, automated pupillometry measurements correlate well with ICP assessed via invasive parenchymal monitoring in severe brain injury, emphasizing their value as a non-invasive neuromonitoring tool in the early phase of brain injury (13). Automated pupillometry has been studied across the injury spectrum, including mild and moderate cases where associations with long-term outcomes have been reported; however, most prior studies have relied on single time-point measurements obtained at hospital arrival or data collected in the ICU, rather than repeated assessments during the hyperacute phase (e.g., during the ED stay) (14). Since prehospital unilateral reactive pupils in TBI, assessed using penlight testing, are maintained in only 58% of patients during early hospitalization, further investigation into the utility of automated pupillometry in the first hours after brain injury is warranted (15).

This study aimed to characterize ultra-early neurological trajectories in patients with acute brain injury requiring ICU admission by analyzing dynamic changes in automated pupillary indices during the hyperacute phase. Specifically, we investigated the association between ultra-early automated pupillometry measurements (from ED arrival up to 24 h) and patient outcomes. We further sought to examine the predictive value of Neurological Pupil Index (NPi) trends within the first 24 h after ED arrival (NPi trajectories) for clinical outcomes in acute brain injury.

## Methods

### Patient selection

This study utilized data from the Workup on Awakeness for Koma Evaluation, Unicenter registry (WAKE-Up), an ED-based cohort designed to systematically collect pupillometry data in consecutive patients presenting with altered level of consciousness at a tertiary referral hospital (16).

Patients aged 18 years or older who presented to the ED between September 1, 2022, and July 31, 2024, and were diagnosed with acute traumatic brain injury, intracerebral hemorrhage (with or without intraventricular hemorrhage), aneurysmal subarachnoid hemorrhage, or ischemic stroke requiring ICU admission were identified from the registry and included in the analysis. This study was approved by the institutional review board of Seoul National University Bundang Hospital (approval number: B-2205-757-303 and B-2402-885-104) and conducted in accordance with the institutional ethical standards and with the Helsinki Declaration of 1975. The requirement for written informed consent was waived by the institutional review board due to the retrospective nature of the study.

### Clinical data collection

Clinical data, including demographics, medical history (hypertension, diabetes mellitus, stroke, chronic kidney disease, malignancy, smoking history, prior antiplatelet agent use, and prior anticoagulant use), and the type and severity of acute brain injury were collected through a review of medical records. Baseline severity of the injury was assessed using the Glasgow Coma Scale (GCS) upon arrival and pre-injury status and 6-month functional status were assessed using the modified Rankin Scale (mRS).

### Neurological pupillary index

Automated infrared pupillometry was performed using NPi®-200 or NPi®-300 devices (NeurOptics, Irvine, CA). These systems deliver a standardized light stimulus of fixed intensity and duration and incorporate automated ambient light compensation using an infrared detection system. The NPi is a composite score (range 0–5) derived from multiple parameters of the pupillary light reflex, including latency, constriction velocity, dilation velocity, and percent constriction, which incorporates baseline pupil diameter into the dynamic response profile (17). All measurements were obtained using the manufacturer-provided eyecup, which minimizes external light interference and ensures consistent positioning. Assessments were performed by trained personnel according to a standardized protocol. Because automated pupillometry does not require active patient participation, measurements were feasible across varying levels of consciousness.

For this study, indices derived from automated pupillometry—including the NPi, quantitative pupillary light reflex (qPLR), constriction velocity (CV), and dilation velocity (DV)—were analyzed. An NPi ≥ 3 was considered normal, and 0 indicated non-reactivity. The qPLR is defined as the difference in pupil size between baseline and post-stimulation, expressed as a percentage (18). Automated pupillometry assessments were performed immediately upon ED arrival and at 1, 3, 6, and 24 h after ED arrival, according to a predefined study protocol, with data prospectively collected as part of the WAKE-Up registry. Pupillometry assessments performed in the ED were recorded on dedicated case report forms and were not entered into the electronic health record or reported to the treating teams in real time. For the analyses, the lowest values recorded from both eyes at each time point were utilized, as continuous variables (14).

### Outcomes

The primary outcome of the study was ICU mortality. The secondary outcomes included 6-month mortality and poor functional outcome (mRS 4–6) at 6 months. Data on outcomes were prospectively collected through routine outpatient clinic visits or via telephone interviews with patient or family members 6 months post-injury.

### Statistical analysis

Baseline characteristics were compared and summarized using *χ*^2^ tests for categorical variables and *t*-tests or Kruskal-Wallis tests for continuous variables, as appropriate. Continuous variables are presented as mean ± standard deviation (SD) or median with interquartile range (IQR), as appropriate, and categorical variables as counts with percentages. Associations between pupillometry indices at each time point (0, 1, 3, 6, 24 h) as continuous variables and outcomes were assessed using multivariable logistic regression analyses, adjusted for age, sex, initial GCS score, type of index injury, and time from symptom onset to ED arrival (14, 18). As a sensitivity analysis, the effect of pupillary indices on outcomes was investigated in patients who did not undergo surgical intervention within 24 h of arrival. Additionally, associations between dichotomized NPi values (<3 vs. ≥3) and outcomes were investigated. Results of the regression models were reported as unadjusted and adjusted odds ratios (OR) with 95% confidence intervals (CIs).

Considering the dynamic change in NPi values, NPi trajectories within 24 h of ED arrival were clustered into four groups using k-means longitudinal clustering, an unsupervised clustering method for time series data (19). Group assignment was determined based on overall similarity of longitudinal NPi profiles across the entire 24-h period, without dichotomization or threshold transformation (e.g., NPi = 3). The optimal number of clusters (k) was explored between 2 and 6, and the final number of clusters was selected based on the Calinski–Harabasz criterion (20). Associations between NPi trajectory groups and outcomes were evaluated using multivariable logistic regression models. To compare the predictive value of the pupillometry indices, Receiver-Operating Characteristic (ROC) curves and Area Under the Curve (AUC) statistics were employed. The performance of models predicting primary and secondary outcomes (Model 1, clinical variables including age, sex, initial GCS, injury type, and time from onset to ED arrival; Model 2, Model 1 + NPi at arrival; Model 3, Model 1 + NPi at 24 h; Model 4, Model 1 + NPi trajectories) was compared using DeLong’s method (21). All statistical analyses were performed using R version 4.3.1 (R Development Core Team, Vienna, Austria). All *p*-values were 2-sided, with *p* < 0.05 considered statistically significant, and without adjustments for multiple testing due to the nature of a small proof-of-concept study.

## Results

### Patient characteristics

Among 1,064 patients prospectively enrolled in the WAKE-Up registry during the study period, 292 were diagnosed with acute brain injury. Of these, 168 patients required neuro-ICU admission and were included in the present analysis. The diagnostic distribution of excluded patients is presented in Supplementary Table 1. The mean age of the patients was 69.6 ± 15.2 years, and 91 (54.5%) were male. Baseline characteristics stratified by sex are provided in Supplementary Table 2. In the final cohort, 65 patients (38.9%) were diagnosed with ICH with or without IVH, 29 (17.4%) with ischemic stroke, 20 (12.0%) with SAH, and 53 (31.7%) with TBI. The median GCS score upon ED arrival was 7 [5–9]. The median time from onset to ED arrival was 1.6 [0.9–5.2] h, from ED arrival to ICU admission was 5.0 [3.5–6.4] h, and from onset to ICU admission was 7.4 [5.5–12.8] h. Furthermore, 47 patients (28.1%) underwent surgical intervention within 24 h of arrival.

### Ultra-early automated pupillometry assessments

A total of 1,504 automated pupillometry assessments from both eyes of the 168 patients were included in the analysis. Pupillometry indices were available for 167 patients at ED arrival, 155 patients at 1 h from ED arrival, 120 patients at 3 h, 157 patients at 6 h, and 153 patients at 24 h after ED arrival. The causes of missing pupillometry measurements at each time point are summarized in Supplementary Table 3. The median number of pupillometry assessments conducted during the ED stay was 3 [2–3], and 2 [1–2] after ICU admission.

The median NPi values at each time point were 3.6 [0.0–4.5] at ED arrival, 3.4 [0.0–4.4] at 1 h, 3.7 [0.0–4.5] at 3 h, 3.8 [0.0–4.5] at 6 h, and 3.9 [0.0–4.5] at 24 h. The number of patients with an NPi < 3 was 65 (38.9%) at ED arrival, 69 (44.5%) at 1 h, 47 (39.2%) at 3 h, 62 (39.5%) at 6 h, and 50 (32.7%) at 24 h. Patients with an NPi < 3 on arrival had a higher proportion of TBI (47.7% vs. 21.6%), lower proportion of ICH (32.3% vs. 43.1%), and a lower median GCS score [5 (3–7) vs. 9 (7–11)], compared to those with NPi ≥ 3 (Table 1).

**Table 1:** Patient characteristics by pupil reactivity at ED arrival.

Characteristic	Overall^a^	NPi ≥ 3 (*n* = 102)	NPi < 3 (*n* = 65)	*p*-value
Age, years, mean ± SD	69.6 ± 15.2	70.6 ± 14.2	68.1 ± 16.7	0.33
Male, *n* (%)	91 (54.5%)	52 (51.0%)	39 (60.0%)	0.33
Medical history, *n* (%)				
Hypertension	91 (54.5%)	57 (55.9%)	34 (52.3%)	0.77
Diabetes	61 (36.5%)	39 (38.2%)	22 (33.8%)	0.68
Stroke	49 (29.3%)	28 (27.5%)	21 (32.3%)	0.62
Chronic kidney disease	20 (12.0%)	8 (7.84%)	12 (18.5%)	0.07
Malignancy	23 (13.8%)	14 (13.7%)	9 (13.8%)	0.99
Smoking	37 (22.2%)	25 (24.5%)	12 (18.5%)	0.47
Prior antiplatelet agents, *n* (%)	50 (29.9%)	30 (29.4%)	20 (30.8%)	0.99
Prior anticoagulants, *n* (%)	15 (8.98%)	8 (7.84%)	7 (10.8%)	0.71
Premorbid mRS 0–1, *n* (%)	121 (72.5%)	72 (70.6%)	49 (75.4%)	0.62
Time from onset to arrival, median (h) [IQR]	1.6 [0.9–5.2]	1.8 [0.9–6.7]	1.3 [0.8–4.4]	0.29
Time from arrival to ICU admission, median (h) [IQR]	5.0 [3.5–6.4]	5.0 [3.4–6.6]	5.0 [3.7–5.9]	0.97
Time from onset to ICU admission, median (h) [IQR]	7.4 [5.5–12.8]	7.6 [5.4–13.1]	7.1 [5.7–11.4]	0.64
Injury type, *n* (%)				<0.01
ICH with/without IVH	65 (38.9%)	44 (43.1%)	21 (32.3%)	
Ischemic stroke	29 (17.4%)	24 (23.5%)	5 (7.69%)	
SAH	20 (12.0%)	12 (11.8%)	8 (12.3%)	
TBI	53 (31.7%)	22 (21.6%)	31 (47.7%)	
Initial GCS, median [IQR]	7 [5–9]	9 [7–11]	5 [3–7]	<0.01
Initial GCS, trichotomized, *n* (%)				<0.01
3–5	57 (34.1%)	18 (17.6%)	39 (60.0%)	
6–8	46 (27.5%)	30 (29.4%)	16 (24.6%)	
9–15	64 (38.3%)	54 (52.9%)	10 (15.4%)	
Surgical treatment within 24 h, *n* (%)	47 (28.1%)	31 (30.4%)	16 (24.6%)	0.53

ED, emergency department; NPi, neurological pupil index; ICH, intracerebral hemorrhage; IVH, intraventricular hemorrhage; SAH, subarachnoid hemorrhage; TBI, traumatic brain injury; GCS, Glasgow Coma Scale; SD, standard deviation; IQR, interquartile range.

a Excluding 1 patient with no pupillometry assessment on arrival.

Among patients with an NPi < 3 at each time point, 7 (11.1%) at arrival, 5 (10.9%) at 1 h, 3 (7.0%) at 3 h, and 9 (16.7%) at 6 h showed an improvement in NPi to ≥ 3 at their next NPi assessment. Conversely, of the patients with NPi ≥ 3 at each time point, 12 (13.2%) at arrival, 5 (8.1%) at 1 h, 4 (5.6%) at 3 h, and 3 (3.2%) at 6 h showed a decline to NPi < 3 at the subsequent assessment. The change in NPi values within 24 h is illustrated in Supplementary Figure 1.

### Ultra-early automated pupillometry indices and outcomes

**Figure 1:**
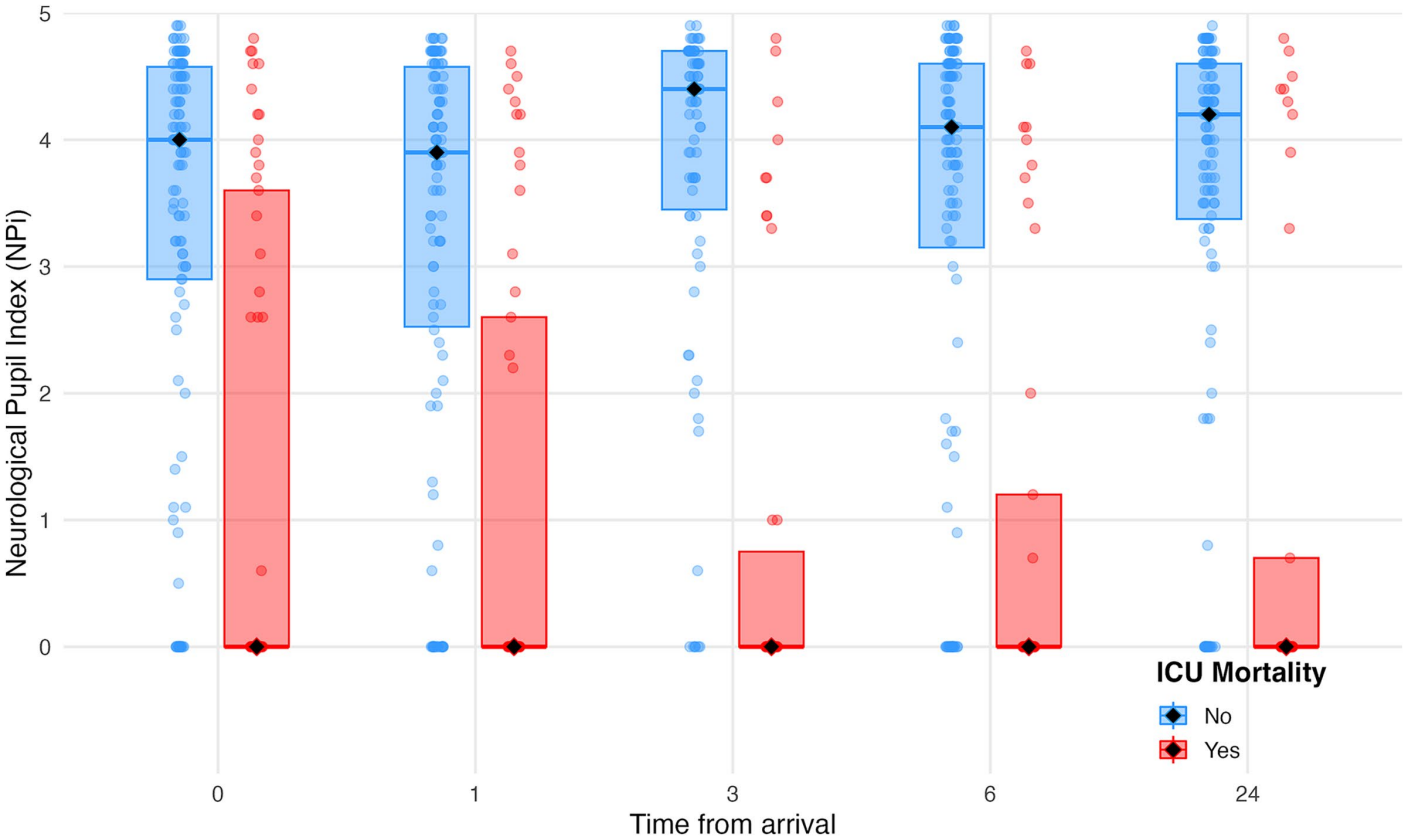
Temporal changes in neurological pupil index within 24 h of arrival according to ICU mortality. Box plots show the distribution of NPi values at each time point stratified by ICU mortality. The boxes represent the interquartile range (IQR), with the horizontal line indicating the median. Individual points represent patient-level measurements. Black diamonds denote the median NPi value for each group at each time point.

Of the patients, 50 (29.8%) died during ICU care. Among those with NPi ≥ 3 on arrival, 15 (14.7%) died during ICU care, while 31 (47.7%) of patients with NPi < 3 on arrival survived. Patients who did not survive had lower initial GCS scores at arrival, received less surgical treatment within the first 24 h, and more frequently exhibited an NPi < 3 at all time points (Supplementary Table 4). The distributions of NPi at each time point according to outcomes are illustrated in Figure 1; Supplementary Figures 2–4.

In multivariable logistic regression analyses, higher NPi values, as a continuous variable, at each time point were significantly associated with a lower ICU mortality [adjusted OR (95% CI): at ED arrival, 0.64 (0.50–0.81); at 1 h, 0.57 (0.43–0.73); at 3 h, 0.29 (0.16–0.46); at 6 h, 0.51 (0.37–0.66); at 24 h, 0.43 (0.30–0.59)] (Table 2). Other automated pupillometry indices including qPLRs, CVs, and DVs at each time point were also associated with ICU mortality (Supplementary Tables 5–7). Dichotomized NPi values (<3 vs. ≥3) at each time point consistently correlated with outcomes (Supplementary Table 8). Similar associations were observed in a sensitivity analysis using NPi values from the eye ipsilateral to the radiologically dominant lesion side (Supplementary Table 9). Among patients who did not receive surgical intervention within 24 h of arrival (*n* = 120), higher NPi values at each time point were likewise associated with lower ICU mortality (Supplementary Table 10).

**Table 2:** Neurological pupil index at each time point and clinical outcome.

Outcome/time point	Crude OR	*p*-value	Adjusted OR^a^	*p*-value
ICU mortality				
NPi at 0 h (*n* = 167)	0.59 [0.49–0.71]	<0.01	0.64 [0.50–0.81]	<0.01
NPi at 1 h (*n* = 155)	0.54 [0.44–0.66]	<0.01	0.57 [0.43–0.73]	<0.01
NPi at 3 h (*n* = 120)	0.42 [0.32–0.54]	<0.01	0.29 [0.16–0.46]	<0.01
NPi at 6 h (*n* = 157)	0.52 [0.41–0.63]	<0.01	0.51 [0.37–0.66]	<0.01
NPi at 24 h (*n* = 153)	0.50 [0.40–0.62]	<0.01	0.43 [0.30–0.59]	<0.01
Mortality at 6 months				
NPi at 0 h (*n* = 149)	0.69 [0.57–0.83]	<0.01	0.74 [0.58–0.93]	0.01
NPi at 1 h (*n* = 139)	0.68 [0.56–0.82]	<0.01	0.74 [0.57–0.95]	0.02
NPi at 3 h (*n* = 106)	0.49 [0.37–0.62]	<0.01	0.31 [0.16–0.50]	<0.01
NPi at 6 h (*n* = 141)	0.63 [0.52–0.76]	<0.01	0.60 [0.47–0.77]	<0.01
NPi at 24 h (*n* = 135)	0.64 [0.52–0.77]	<0.01	0.58 [0.44–0.76]	<0.01
Poor functional outcome at 6 months				
NPi at 0 h (*n* = 149)	0.57 [0.40–0.75]	<0.01	0.54 [0.34–0.79]	<0.01
NPi at 1 h (*n* = 139)	0.52 [0.36–0.69]	<0.01	0.47 [0.28–0.72]	<0.01
NPi at 3 h (*n* = 106)	0.54 [0.35–0.75]	<0.01	0.36 [0.16–0.65]	<0.01
NPi at 6 h (*n* = 141)	0.59 [0.43–0.76]	<0.01	0.58 [0.39–0.80]	<0.01
NPi at 24 h (*n* = 135)	0.62 [0.45–0.81]	<0.01	0.62 [0.41–0.89]	0.01

OR, odds ratio; NPi, neurological pupil index.

a Multivariable models were adjusted for age, sex, initial GCS score, type of index injury, and time from onset to arrival.

At 6-month follow-up, 116 (77.3%) of the 150 patients with available outcome data had a mRS score of 4–6, and 70 (46.7%) had died. In multivariable models, lower automated pupillometry indices (NPi, qPLR, CV, and DV) were associated with both increased 6-month mortality and poor functional outcomes.

### Predictive value of NPi trajectories

**Figure 2:**
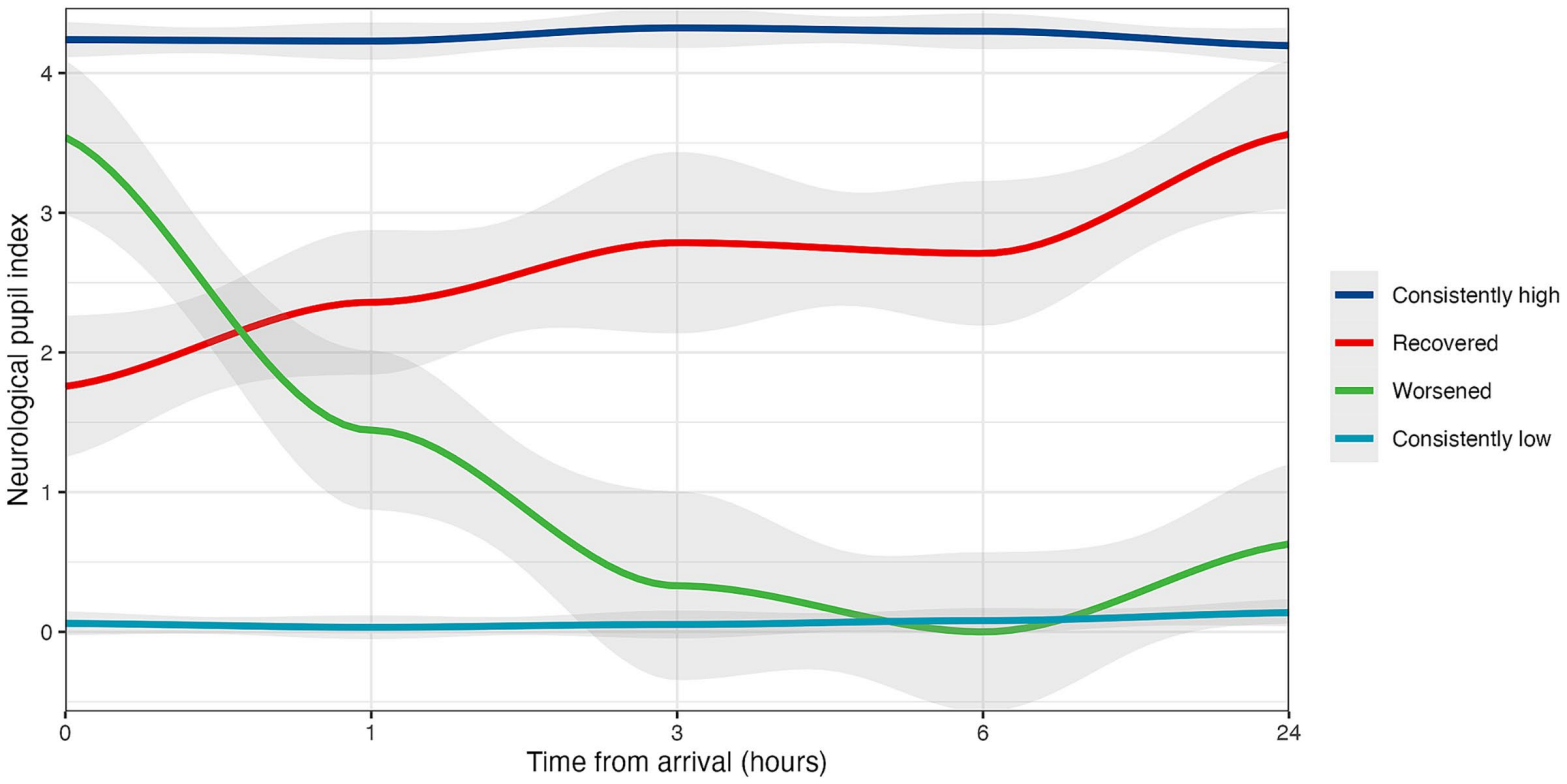
Longitudinal K-means clustering of neurological pupil index (NPi) and identification of distinct NPi trajectories. Colored lines represent LOESS-smoothed trajectories within each k-means–derived cluster. Shaded areas indicate 95% confidence intervals.

Using k-means longitudinal clustering, the patients were categorized into four NPi trajectory groups (Figure 2): “*Consistently high*” (n = 87), “*Recovered*” (*n* = 23), “*Worsened*” (*n* = 15), and “*Consistently low*” (*n* = 42). The median baseline-to-24-h change in NPi was 0 [IQR, −0.3 to 0.3] in the Consistently high group, 1.4 [0.2 to 2.7] in the Recovered group, −3.1 [−3.92 to −2.52] in the Worsened group, and 0 [0 to 0] in the Consistently low group, consistent with their longitudinal patterns. Patient characteristics did not differ significantly across the four trajectory groups, except for a higher proportion of TBIs in the *Recovered* group and a higher proportion of ICHs in the *Worsened* group (Supplementary Table 11).

Compared to the *Consistently high* group, both the *Worsened* and *Consistently low* groups were significantly associated with increased ICU mortality [adjusted OR (95% CI): *Worsened* group, 34.85 (7.59–201.46); *Consistently low* group, 27.91 (7.46–127.06)], 6-month mortality [*Worsened* group, 14.83 (3.43–85.35); *Consistently low* group, 8.94 (2.70–33.60)], and poor functional outcomes [*Worsened* group, 8.65 (1.28–201.46); *Consistently low* group, 22.11 (2.81–511.92)] (Table 3). In contrast, the *Recovered* group was not significantly associated with increased probability of the outcomes compared to the *Consistently high* group [1.12 (0.15–5.59) for ICU mortality, 0.64 (0.15–2.32) for 6-month mortality, and 1.08 (0.27–4.43) for poor functional outcome]. Subgroup analyses stratified by injury type showed no significant interaction between trajectory clusters and underlying etiology (p for interaction = 0.99). Since patients were admitted to the ICU at a median of 5 h after arrival, NPi trajectories were further grouped based on values measured within the first 3 h to explore the association between NPi values in the ED and outcomes. This analysis demonstrated results consistent with the primary findings (Supplementary Table 12).

**Table 3:** Neurological pupil index trajectory groups within 24 h and clinical outcomes.

Outcome/trajectory group	Crude OR	*p*-value	Adjusted OR^a^	*p*-value
ICU mortality				
Group 1: consistently high	Reference	–	Reference	
Group 2: recovered	1.48 [0.30–5.66]	0.59	1.12 [0.15–5.59]	0.90
Group 3: worsened	19.75 [5.68–78.60]	<0.01	34.85 [7.59–201.46]	<0.01
Group 4: consistently low	22.03 [8.67–62.17]	<0.01	27.91 [7.46–127.06]	<0.01
Mortality at 6 months				
Group 1: consistently high	Reference	–	Reference	
Group 2: recovered	0.90 [0.26–2.69]	0.86	0.64 [0.15–2.32]	0.51
Group 3: worsened	10.10 [2.87–47.68]	<0.01	14.83 [3.43–85.35]	<0.01
Group 4: consistently low	7.82 [3.37–19.53]	<0.01	8.94 [2.70–33.60]	<0.01
Poor functional outcome at 6 months				
Group 1: consistently high	Reference	–	Reference	
Group 2: recovered	1.85 [0.41–3.67]	0.77	1.08 [0.27–4.43]	0.92
Group 3: worsened	7.58 [1.40–141.24]	0.06	8.65 [1.28–201.46]	0.06
Group 4: consistently low	21.67 [4.30–395.22]	<0.01	22.11 [2.81–511.92]	0.01

OR, odds ratio.

a Multivariable models were adjusted for age, sex, initial GCS score, type of index injury, and time from onset to arrival.

**Figure 3:**
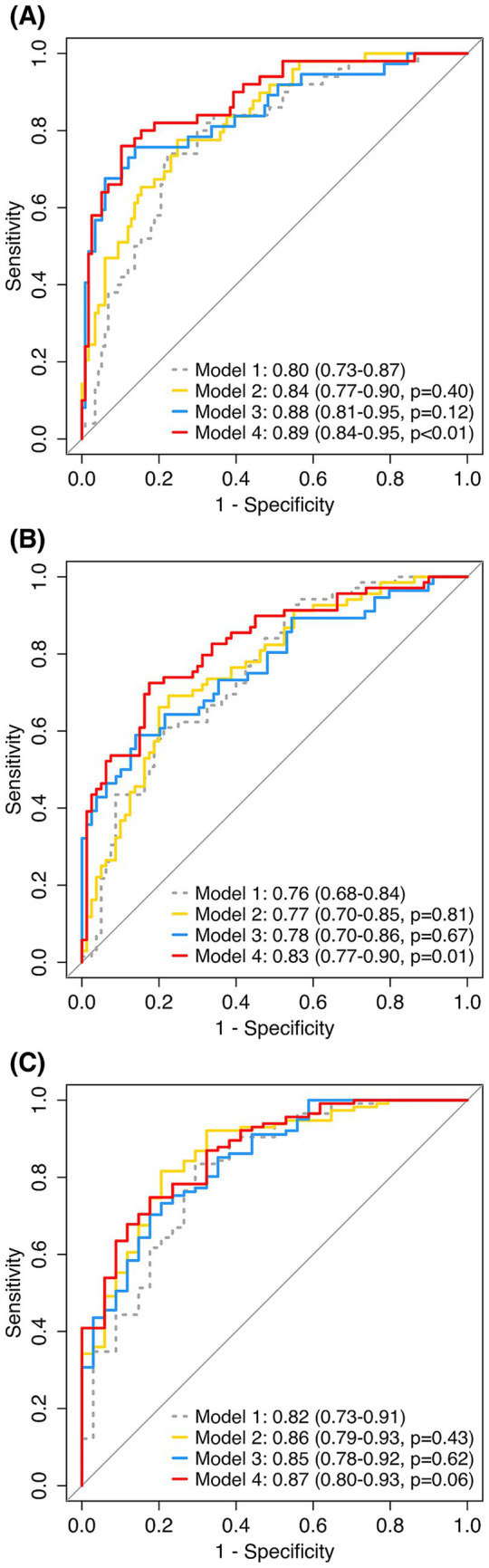
Performance of prediction models with clinical variables, neurological pupil index at 0 h, 24 h, and trajectories: **(A)** ICU mortality; **(B)** 6-month mortality; **(C)** 6-month poor functional outcome. Model 1: clinical variables including age, sex, initial GCS, injury type, and time from onset to ED arrival; Model 2: Model 1 + NPi at arrival; Model 3: Model 1 + NPi at 24 h; Model 4, Model 1 + NPi trajectories. mRS, modified Rankin Scale.

When compared to the prediction models including only clinical variables [AUROC 0.80 (0.73–0.87) for ICU mortality; 0.76 (0.68–0.84) for 6-month mortality; 0.82 (0.73–0.91) for poor functional outcome], the models that additionally included NPi trajectory improved the predictive performance of the model [AUROC 0.89 (0.84–0.95), *p* < 0.01 for ICU mortality; 0.83 (0.77–0.90), *p* = 0.01 for 6-month mortality; 0.87 (0.80–0.93), *p* = 0.06 for poor functional outcome] (Figure 3). However, the addition of NPi values at ED arrival or at 24 h did not significantly improve the performance of the prediction models. Supplementary Table 13 presents the performance metrics of the prediction models.

## Discussion

This study demonstrated that automated pupillometry-derived indices measured within the first 24 h after ED arrival exhibit dynamic changes and are associated with clinical outcomes in patients with acute brain injury. Notably, the *Consistently low* or the *Worsened* ultra-early NPi trajectory groups within 24 h were associated with poor clinical outcomes, while the *Recovered* group showed no significant difference compared to the *Consistently high* group. Although overall mortality was high, outcomes differed markedly across NPi trajectory groups, suggesting heterogeneity in early neurological evolution within this severely ill population. Incorporating NPi trajectories during the hyperacute phase into the prediction model, alongside clinical variables, improved the predictive value.

The association between ultra-early NPi values at each time point and clinical outcomes in patients with acute brain injury observed in this study aligns with previous studies. The ORANGE study showed that brain-injured patients with poor outcome were more likely to have at least one abnormal automated pupillometry measurement from ICU admission until day 7 (without the data from ED), and their median NPi values were significantly lower compared to those with favorable outcomes (14). Similarly, prior investigations have demonstrated that lower NPi values correlate with intracranial hypertension, neurological deterioration, and unfavorable neurological outcomes in severe brain injury populations (22–25). Our findings extend this literature by focusing specifically on repeated ultra-early measurements obtained during the ED stay, thereby emphasizing early neurological dynamics rather than single time-point assessments. Notably, our study demonstrated that initial NPi values at ED arrival—a unique data in our study obtained early after onset (median 1.6 h) and prior to treatment initiation—are independently associated with patient outcomes. While initial NPi values at ED arrival may primarily reflect the severity of brain injury, the consistent association between repeated pupillometry measurements during the ED stay (median of three per patient) and clinical outcomes in our study highlights their value as reliable metrics for early neurological assessment.

Differences in prognosis based on NPi trajectories in our study highlight the importance of repeated NPi measurements during the early phase of injury. Prior research on comatose cardiac arrest survivors has demonstrated that NPi values between favorable and unfavorable outcome groups are closely clustered at 0 and 24 h, diverging significantly after 48 h (18). A study on acute brain injury also determined that two consecutive NPi measurements of 0 significantly increase mortality risk, while recovery of NPi from 0 to higher values in subsequent assessments does not increase mortality (14). In cardiac arrest survivors, the loss of pupillary light reflex, resulting from dysfunction of the relatively anoxia-resistant midbrain, indicates global ischemic damage that is rarely reversible (26). In contrast, neurological deterioration in acute brain injury, including the loss of pupillary light reflex, is often reversible with prompt intervention. Furthermore, changes in pupillary light reflex may precede clinical deterioration or radiographic evidence of midline shift, offering an opportunity for timely intervention to mitigate progression of injury (27, 28). Moreover, since the benefit of invasive ICP monitoring in brain-injured patients is maximized when applied in patient with at least one unresponsive pupillary light reflex, early automated pupillometry measurements in the ED may provide a reliable standard for determining treatment intensity and neuroimaging follow-up during the hyperacute phase (29).

In our study, the most common injury type differed according to the NPi trajectory group: TBI was most common in the *Recovered* trajectory group, while ICH was more frequent in the *Worsened* trajectory group. The higher prevalence of TBI in the *Recovered* group may be attributed to the resolution of transient neurological dysfunction following concussion (30). Additionally, this finding may be explained by the effect of early hematoma evacuation for acute subdural hemorrhages, with 24/7 availability at the study center. Conversely, *Worsened* NPi in ICH patients may be due to hematoma expansion, which often occurs within the first 6 h (31–33). In contrast, ischemic stroke was more frequently represented in the Consistently high trajectory group, suggesting that pupillary abnormalities may be less prominent during the first 24 h unless malignant edema, hemorrhagic transformation, or posterior circulation involvement develops. In aneurysmal SAH, early NPi abnormalities may reflect acute hydrocephalus or early intracranial pressure elevation, whereas delayed cerebral ischemia and vasospasm typically occur in the subsequent days. Although these observations should be interpreted cautiously given the limited sample size, they suggest that ultra-early NPi trajectories may reflect injury-specific pathophysiological patterns and support further investigation of trajectory changes in the subacute window, particularly from 24 h to 7 days.

This study has several limitations. First, as a retrospective single-center study, the findings may not be generalizable to other populations or healthcare settings. Second, since we focused on pupillometry measurements within the first 24 h of ED arrival, changes in neurological status beyond this timeframe may have influenced patient prognosis. Third, missing data at certain time points, particularly 3 h after ED arrival, likely corresponding to the timing of emergent surgical interventions, may have affected the robustness of our findings. However, consistency in non-surgical subgroups supports their validity. Fourth, although pupillometry assessments performed in the ED were blinded to treating clinicians and recorded only on study case report forms, pupillometry was sometimes documented in parallel by bedside nurses after ICU admission as part of routine monitoring. Thus, while management decisions in the ED were independent of study pupillometry data, the possibility of partial unblinding during the ICU phase cannot be excluded, and subsequent ICU pupillometry, together with the overall clinical course, may have influenced treatment intensity, including timely interventions or early de-escalation. Fifth, the study lacked detailed information on other potential confounders, such as pre-hospital care, eyelid handling in unconscious patients, or family decisions regarding life-sustaining treatments, which could have influenced outcomes. Finally, despite the use of multivariable analyses, the possibility of residual confounding cannot be excluded.

## Conclusion

Our study demonstrated that ultra-early automated pupillometry indices exhibit dynamic changes within the first 24 h of ED arrival, with these early NPi changes significantly associated with clinical outcomes in patients with acute brain injury. *Worsened* or *Consistently low* NPi trajectories were associated with poor outcomes, whereas a *Recovered* NPi trajectory was not. Incorporating NPi trajectories improved the performance of the outcome prediction model compared to a model based on clinical variables. Future prospective studies are needed to validate the utility of automated pupillometry in the ED and to explore its role in early neurological monitoring and risk stratification in acute brain injury.

## Data Availability

De-identified participant data analyzed in this study can be made available to qualified investigators upon reasonable request to the corresponding author. Requests to access these datasets should be directed to Dong Keon Lee, stolenegg@gmail.com.
