# Supplementary material for: Ultra-early neurological pupil index trajectories and clinical outcomes in acute brain injury
Source: Front Neurol. 2026 Jun 12;17:1741572. doi: 10.3389/fneur.2026.1741572 (PMC13303215; doi:10.3389/fneur.2026.1741572)

**Supplemental Material**

**Ultra-early Neurological Pupil Index Trajectories and Clinical Outcomes in Acute Brain Injury**

**Table of contents**

Supplemental Table 1. Etiologies of altered level of consciousness in excluded patients

Supplemental Table 2. Baseline Characteristics Stratified by Sex

Supplemental Table 3. Causes of missing NPi assessments (by time point)

Supplemental Table 4. Patient characteristics according to ICU mortality

Supplemental Table 5. Quantitative Pupil Light Reflex at each time point and outcomes

Supplemental Table 6. Constriction velocity at each time point and outcomes

Supplemental Table 7. Dilation velocity at each time point and outcomes

Supplemental Table 8. Dichotomized Neurological Pupillary index (<3.0) and outcomes

Supplemental Table 9. Sensitivity analysis using ipsilateral NPi values relative to lesion side

Supplemental Table 10. Neurological Pupillary index at each time point and outcome in patients without surgical intervention within 24 hours

Supplemental Table 11. Patient characteristics according to Neurological Pupillary index trajectories

Supplemental Table 12. Neurological Pupil index trajectories within 3 hours and outcome

Supplemental Table 13. Performance metrics of the prediction models

Supplemental Figure 1. Changes in Neurological Pupil index (NPi) over 24 hours

Supplemental Figure 2. Changes in Neurological pupil index (NPi) over time stratified by ICU mortality

Supplemental Figure 3. Changes in Neurological pupil index (NPi) over time stratified by mortality at 6 months

Supplemental Figure 4. Changes in Neurological pupil index (NPi) over time stratified by mRS at 6 months

Supplemental Table 1. Etiologies of altered level of consciousness in excluded patients

|  | Patient number (%) |
| --- | --- |
| Brain hemorrhage | 51 (5.7%) |
| Brain tumor | 27 (3.0%) |
| CNS infection | 11 (1.2%) |
| Drug | 177 (19.8%) |
| Epilepsy | 52 (5.8%) |
| Ischemic stroke | 73 (8.1%) |
| Metabolic encephalopathy | 460 (51.3%) |
| Functional disorder | 13 (1.5%) |
| Others | 32 (3.5%) |

Supplemental Table 2. Baseline Characteristics Stratified by Sex

|  | Female (n=76) | Male (n=92) | *p*-value |
| --- | --- | --- | --- |
| Age, years, mean ± SD | 72.8 ± 15.0 | 66.9 ± 14.9 | 0.01 |
| Medical history, n (%) |  |  |  |
| Hypertension | 43 (56.6%) | 49 (53.3%) | 0.78 |
| Diabetes | 27 (35.5%) | 34 (37.0%) | 0.98 |
| Stroke | 26 (34.2%) | 23 (25.0%) | 0.26 |
| Chronic kidney disease | 9 (11.8%) | 11 (12.0%) | 0.99 |
| Malignancy | 12 (15.8%) | 11 (12.0%) | 0.62 |
| Smoking | 6 (7.9%) | 31 (33.7%) | <0.01 |
| Prior antiplatelet agents, n (%) | 23 (30.3%) | 27 (29.3%) | 0.99 |
| Prior anticoagulants, n (%) | 9 (11.8%) | 6 (6.5%) | 0.35 |
| Premorbid mRS 0-1, n (%) | 52 (68.4%) | 70 (76.1%) | 0.35 |
| Time from onset to arrival, median (hours) [IQR] | 1.2 [0.8–3.3] | 2.1 [1.0–6.8] | 0.06 |
| Time from arrival to ICU admission, median (hours) [IQR] | 4.7 [3.3–5.7] | 5.1 [3.6–7.6] | 0.13 |
| Time from onset to ICU admission, median (hours) [IQR] | 6.3 [4.9–9.6] | 8.7 [6.2–13.6] | <0.01 |
| Injury type, n (%) |  |  | <0.01 |
| ICH with/without IVH | 36 (47.4%) | 30 (32.6%) |  |
| Ischemic stroke | 14 (18.4%) | 15 (16.3%) |  |
| SAH | 14 (18.4%) | 6 (6.5%) |  |
| TBI | 12 (15.8%) | 41 (44.6%) |  |
| Initial GCS, median [IQR] | 7 [5–10] | 7 [4–9] | 0.46 |
| Initial GCS, trichotomized, n (%) |  |  | 0.62 |
| 3-5 | 24 (31.6%) | 33 (35.9%) |  |
| 6-8 | 20 (26.3%) | 27 (29.3%) |  |
| 9-15 | 32 (42.1%) | 32 (34.8%) |  |
| Surgical treatment within 24h, n (%) | 25 (32.9%) | 22 (23.9%) | 0.26 |

SD, standard deviation; mRS, modified Rankin Scale; IQR, interquartile range; ICU, intensive care unit; ICH, intracerebral hemorrhage; IVH, intraventricular hemorrhage; SAH, subarachnoid hemorrhage; TBI, traumatic brain injury; GCS, Glasgow Coma Scale.

Supplemental Table 3. Causes of missing NPi assessments (by time point)

|  | Missing cause | Patient number (%) |
| --- | --- | --- |
| Overall | Surgery | 43 (48.9%) |
|  | Neurointervention | 11 (12.5%) |
|  | Death | 8 (9.1%) |
|  | Unreliable measure | 7 (8.0%) |
|  | Imaging study | 5 (5.7%) |
|  | COVID-19 | 3 (3.4%) |
|  | Others | 4 (4.5%) |
|  | Not recorded | 7 (8.0%) |
| 0h | Unreliable measure | 1 (100%) |
| 1h | Neurointervention | 6 (46.2%) |
|  | Imaging study | 4 (30.8%) |
|  | Unreliable measure | 2 (15.4%) |
|  | Surgery | 1 (7.7%) |
| 3h | Surgery | 36 (75.0%) |
|  | Neurointervention | 5 (10.4%) |
|  | COVID-19 | 1 (2.1%) |
|  | Imaging study | 1 (2.1%) |
|  | Unreliable measure | 1 (2.1%) |
|  | Others | 2 (4.2%) |
|  | Not recorded | 2 (4.2%) |
| 6h | Surgery | 6 (54.5%) |
|  | COVID-19 | 1 (9.1%) |
|  | Death | 1 (9.1%) |
|  | Unreliable measure | 1 (9.1%) |
|  | Others | 1 (9.1%) |
|  | Not recorded | 1 (9.1%) |
| 24h | Death | 7 (46.7%) |
|  | Unreliable measure | 2 (13.3%) |
|  | COVID-19 | 1 (6.7%) |
|  | Others | 1 (6.7%) |
|  | Not recorded | 4 (26.7%) |

Supplemental Table 4. Patient characteristics according to ICU mortality

|  | Survivors (n=118) | Non-survivors (n=50) | p-value |
| --- | --- | --- | --- |
| Age | 69.1 ± 15.1 | 70.7 ± 15.5 | 0.53 |
| Male | 61 (51.7%) | 31 (62.0%) | 0.29 |
| Medical history |  |  |  |
| Hypertension | 62 (52.5%) | 30 (60.0%) | 0.47 |
| Diabetes | 42 (35.6%) | 19 (38.0%) | 0.90 |
| Stroke | 30 (25.4%) | 19 (38.0%) | 0.15 |
| Chronic kidney disease | 11 (9.32%) | 9 (18.0%) | 0.18 |
| Malignancy | 15 (12.7%) | 8 (16.0%) | 0.75 |
| Smoking | 27 (22.9%) | 10 (20.0%) | 0.84 |
| Prior antiplatelet agents | 32 (27.1%) | 18 (36.0%) | 0.33 |
| Prior anticoagulants | 9 (7.63%) | 6 (12.0%) | 0.38 |
| Premorbid mRS 0-1 | 85 (72.0%) | 37 (74.0%) | 0.94 |
| Time from onset to arrival (h) | 1.8 [1.0 - 6.1] | 1.1 [0.7 - 3.5] | 0.06 |
| Injury type |  |  | 0.41 |
| ICH/IVH | 44(37.3%) | 22 (44.0%) |  |
| Ischemic stroke | 23(19.5%) | 6 (12.0%) |  |
| SAH | 16(13.6%) | 4 (8.0%) |  |
| TBI | 35(29.7%) | 18 (36.0%) |  |
| Initial GCS | 9 [6 - 11] | 5 [3 - 7] | <0.01 |
| Initial GCS, trichotomized |  |  | <0.01 |
| 3-5 | 27 (22.9%) | 30 (60.0%) |  |
| 6-8 | 32 (27.1%) | 15 (30.0%) |  |
| 9-15 | 59 (50.0%) | 5 (10.0%) |  |
| Surgical treatment within 24h | 41 (34.7%) | 6 (12.0%) | <0.01 |
| Neurologic Pupillary index < 3 |  |  |  |
| At arrival | 31 (26.3%) | 34 (69.4%) | <0.01 |
| At 1 hour | 31 (29.2%) | 38 (77.6%) | <0.01 |
| At 3 hours | 14 (17.9%) | 33 (78.6%) | <0.01 |
| At 6 hours | 27 (24.1%) | 35 (77.8%) | <0.01 |
| At 24 hours | 22 (19.0%) | 28 (75.7%) | <0.01 |

ICU, intensive care unit; mRS, modified Rankin Scale; ICH, intracerebral hemorrhage; IVH, intraventricular hemorrhage; SAH, subarachnoid hemorrhage; TBI, traumatic brain injury; GCS, Glasgow Coma Scale.

Supplemental Table 5. Quantitative Pupil Light Reflex at each time point and outcomes

| ICU mortality |  |  |  |  |
| --- | --- | --- | --- | --- |
|  | Crude HR | p-value | Adjusted HR* | p-value |
| 0h (n=167) | 0.93 [0.91–0.96] | <0.01 | 0.94 [0.91–0.98] | <0.01 |
| 1h (n=155) | 0.91 [0.87–0.95] | <0.01 | 0.92 [0.88–0.97] | <0.01 |
| 3h (n=120) | 0.84 [0.79–0.90] | <0.01 | 0.83 [0.77–0.90] | <0.01 |
| 6h (n=157) | 0.90 [0.85–0.94] | <0.01 | 0.90 [0.86–0.95] | <0.01 |
| 24h (n=153) | 0.89 [0.84–0.94] | <0.01 | 0.89 [0.84–0.95] | <0.01 |
| Mortality at 6 months |  |  |  |  |
|  | Crude HR | p-value | Adjusted HR* | p-value |
| 0h (n=149) | 0.96 [0.94–0.98] | <0.01 | 0.96 [0.94–0.98] | <0.01 |
| 1h (n=139) | 0.94 [0.92–0.96] | <0.01 | 0.94 [0.92–0.97] | <0.01 |
| 3h (n=106) | 0.90 [0.87–0.93] | <0.01 | 0.89 [0.85–0.92] | <0.01 |
| 6h (n=141) | 0.94 [0.91–0.96] | <0.01 | 0.94 [0.91–0.96] | <0.01 |
| 24h (n=135) | 0.93 [0.91–0.96] | <0.01 | 0.93 [0.90–0.96] | <0.01 |
| Poor functional outcome at 6 months |  |  |  |  |
|  | Crude OR | p-value | Adjusted OR* | p-value |
| 0h (n=149) | 0.94 [0.91–0.97] | <0.01 | 0.94 [0.90–0.98] | <0.01 |
| 1h (n=139) | 0.91 [0.87–0.94] | <0.01 | 0.91 [0.86–0.96] | <0.01 |
| 3h (n=106) | 0.91 [0.86–0.95] | <0.01 | 0.88 [0.81–0.94] | <0.01 |
| 6h (n=141) | 0.94 [0.90–0.97] | <0.01 | 0.90 [0.85–0.95] | <0.01 |
| 24h (n=135) | 0.95 [0.91–0.98] | <0.01 | 0.96 [0.91–1.01] | 0.09 |

HR, hazard ratio; OR, odds ratio; ICU, intensive care unit.

*Multivariable models were adjusted for age, sex, initial GCS score, type of index injury, and time from onset to arrival

Supplemental Table 6. Constriction velocity at each time point and outcomes

| ICU mortality |  |  |  |  |
| --- | --- | --- | --- | --- |
|  | Crude HR | p-value | Adjusted HR* | p-value |
| 0h (n=167) | 0.37 [0.23–0.61] | <0.01 | 0.45 [0.27–0.76] | <0.01 |
| 1h (n=155) | 0.23 [0.12–0.44] | <0.01 | 0.29 [0.14–0.62] | <0.01 |
| 3h (n=120) | 0.05 [0.01–0.18] | <0.01 | 0.04 [0.01–0.19] | <0.01 |
| 6h (n=157) | 0.13 [0.05–0.34] | <0.01 | 0.15 [0.05–0.43] | <0.01 |
| 24h (n=153) | 0.09 [0.03–0.31] | <0.01 | 0.09 [0.02–0.36] | <0.01 |
| Mortality at 6 months |  |  |  |  |
|  | Crude HR | p-value | Adjusted HR* | p-value |
| 0h (n=149) | 0.53 [0.38–0.76] | <0.01 | 0.62 [0.42–0.91] | 0.01 |
| 1h (n=139) | 0.45 [0.31–0.66] | <0.01 | 0.50 [0.33–0.76] | 0.03 |
| 3h (n=106) | 0.24 [0.14–0.43] | <0.01 | 0.22 [0.11–0.42] | <0.01 |
| 6h (n=141) | 0.34 [0.21–0.56] | <0.01 | 0.34 [0.20–0.58] | <0.01 |
| 24h (n=135) | 0.33 [0.19–0.56] | <0.01 | 0.34 [0.19– 0.59] | <0.01 |
| Poor functional outcome at 6 months |  |  |  |  |
|  | Crude OR | p-value | Adjusted OR* | p-value |
| 0h (n=149) | 0.40 [0.24–0.63] | <0.01 | 0.41 [0.22–0.73] | <0.01 |
| 1h (n=139) | 0.32 [0.19–0.52] | <0.01 | 0.39 [0.21–0.71] | <0.01 |
| 3h (n=106) | 0.29 [0.15–0.52] | <0.01 | 0.29 [0.12–0.60] | <0.01 |
| 6h (n=141) | 0.30 [0.17–0.50] | <0.01 | 0.18 [0.08–0.38] | <0.01 |
| 24h (n=135) | 0.47 [0.28–0.77] | <0.01 | 0.52 [0.27–0.998] | 0.049 |

HR, hazard ratio; OR, odds ratio; ICU, intensive care unit.

*Multivariable models were adjusted for age, sex, initial GCS score, type of index injury, and time from onset to arrival

Supplemental Table 7. Dilation velocity at each time point and outcomes

| ICU mortality |  |  |  |  |
| --- | --- | --- | --- | --- |
|  | Crude HR | p-value | Adjusted HR* | p-value |
| 0h (n=166) | 0.08 [0.03–0.25] | <0.01 | 0.10 [0.03–0.36] | <0.01 |
| 1h (n=155) | 0.03 [0.01–0.12] | <0.01 | 0.03 [0.01–0.20] | <0.01 |
| 3h (n=120) | 0.003 [0.0002–0.04] | <0.01 | 0.003 [0.0002–0.04] | <0.01 |
| 6h (n=157) | 0.01 [0.002–0.11] | <0.01 | 0.02 [0.003–0.17] | <0.01 |
| 24h (n=153) | 0.01 [0.002–0.14] | <0.01 | 0.02 [0.002–0.21] | <0.01 |
| Mortality at 6 months |  |  |  |  |
|  | Crude HR | p-value | Adjusted HR* | p-value |
| 0h (n=149) | 0.21 [0.10–0.45] | <0.01 | 0.25 [0.11–0.61] | <0.01 |
| 1h (n=139) | 0.12 [0.04–0.30] | <0.01 | 0.13 [0.04–0.38] | <0.01 |
| 3h (n=106) | 0.07 [0.02–0.20] | <0.01 | 0.04 [0.01–0.14] | <0.01 |
| 6h (n=141) | 0.09 [0.03–0.27] | <0.01 | 0.10 [0.03–0.29] | <0.01 |
| 24h (n=135) | 0.13 [0.05–0.38] | <0.01 | 0.14 [0.05–0.43] | <0.01 |
| Poor functional outcome at 6 months |  |  |  |  |
|  | Crude OR | p-value | Adjusted OR* | p-value |
| 0h (n=149) | 0.14 [0.05–0.39] | <0.01 | 0.16 [0.04–0.60] | <0.01 |
| 1h (n=139) | 0.06 [0.02–0.22] | <0.01 | 0.09 [0.02–0.47] | <0.01 |
| 3h (n=106) | 0.07 [0.01–0.28] | <0.01 | 0.03 [0.003–0.22] | <0.01 |
| 6h (n=141) | 0.08 [0.02–0.25] | <0.01 | 0.02 [0.002–0.11] | <0.01 |
| 24h (n=135) | 0.21 [0.07–0.64] | <0.01 | 0.41 [0.08–1.98] | 0.26 |

HR, hazard ratio; OR, odds ratio; ICU, intensive care unit.

*Multivariable models were adjusted for age, sex, initial GCS score, type of index injury, and time from onset to arrival

Supplemental Table 8. Dichotomized Neurological Pupillary index (<3.0) and outcomes

| ICU mortality |  |  |  |  |
| --- | --- | --- | --- | --- |
|  | Crude HR | p-value | Adjusted HR* | p-value |
| 0h (49 / 167) | 4.56 [2.44–8.51] | <0.01 | 3.19 [1.60–6.37] | <0.01 |
| 1h (49 / 155) | 5.65 [2.81–11.37] | <0.01 | 4.38 [1.92–10.02] | <0.01 |
| 3h (42 / 120) | 9.56 [4.40–20.80] | <0.01 | 8.78 [3.79–20.37] | <0.01 |
| 6h (45 / 157) | 6.92 [3.32–14.40] | <0.01 | 6.05 [2.72–13.46] | <0.01 |
| 24h (37 / 153) | 7.66 [3.49–16.82] | <0.01 | 9.03 [3.62–22.53] | <0.01 |
| Mortality at 6 months |  |  |  |  |
|  | Crude HR | p-value | Adjusted HR* | p-value |
| 0h (69 / 149) | 3.22 [1.98–5.22] | <0.01 | 2.71 [1.55–4.75] | <0.01 |
| 1h (67 / 139) | 3.01 [1.83–4.98] | <0.01 | 2.20 [1.21–4.00] | 0.01 |
| 3h (55 / 106) | 5.08 [2.87–8.98] | <0.01 | 6.68 [3.39–13.14] | <0.01 |
| 6h (63 / 141) | 4.04 [2.40–6.81] | <0.01 | 4.23 [2.34–7.65] | <0.01 |
| 24h (56 / 135) | 4.25 [2.49–7.26] | <0.01 | 6.13 [3.04–12.36] | <0.01 |
| Poor functional outcome at 6 months |  |  |  |  |
|  | Crude OR | p-value | Adjusted OR* | p-value |
| 0h (115 / 149) | 5.32 [2.07–16.49] | <0.01 | 4.71 [1.36–19.18] | 0.02 |
| 1h (107 / 139) | 9.65 [3.49–34.29] | <0.01 | 10.54 [2.61–55.26] | <0.01 |
| 3h (84 / 106) | 4.50 [1.53–16.59] | 0.01 | 7.33 [1.31–57.23] | 0.03 |
| 6h (108 / 141) | 5.81 [2.25–18.11] | <0.01 | 4.83 [1.47–18.26] | 0.01 |
| 24h (101 / 135) | 7.98 [2.63–34.74] | <0.01 | 7.58 [1.76–44.67] | 0.01 |

HR, hazard ratio; OR, odds ratio; ICU, intensive care unit.

*Multivariable models were adjusted for age, sex, initial GCS score, type of index injury, and time from onset to arrival

Supplemental Table 9. Sensitivity analysis using ipsilateral NPi values relative to lesion side

|  | Crude OR | p-value | Adjusted OR^a^ | p-value |
| --- | --- | --- | --- | --- |
| ICU mortality |  |  |  |  |
| NPi at 0h (n=167) | 0.62 [0.51–0.74] | <0.01 | 0.66 [0.51–0.84] | <0.01 |
| NPi at 1h (n=155) | 0.55 [0.45–0.67] | <0.01 | 0.57 [0.44–0.73] | <0.01 |
| NPi at 3h (n=120) | 0.40 [0.30–0.52] | <0.01 | 0.35 [0.21–0.51] | <0.01 |
| NPi at 6h (n=157) | 0.51 [0.41–0.62] | <0.01 | 0.53 [0.39–0.68] | <0.01 |
| NPi at 24h (n=153) | 0.49 [0.39–0.61] | <0.01 | 0.46 [0.33–0.62] | <0.01 |
| Mortality at 6 months |  |  |  |  |
| NPi at 0h (n=149) | 0.71 [0.59–0.85] | <0.01 | 0.78 [0.61–0.99] | 0.04 |
| NPi at 1h (n=139) | 0.70 [0.58–0.83] | <0.01 | 0.75 [0.59–0.96] | 0.02 |
| NPi at 3h (n=106) | 0.47 [0.34–0.60] | <0.01 | 0.40 [0.24–0.59] | <0.01 |
| NPi at 6h (n=141) | 0.64 [0.52–0.76] | <0.01 | 0.65 [0.51–0.82] | <0.01 |
| NPi at 24h (n=135) | 0.64 [0.52–0.77] | <0.01 | 0.62 [0.47–0.80] | <0.01 |
| Poor functional outcome at 6 months |  |  |  |  |
| NPi at 0h (n=149) | 0.62 [0.45–0.81] | <0.01 | 0.58 [0.37–0.85] | 0.01 |
| NPi at 1h (n=139) | 0.59 [0.43–0.76] | <0.01 | 0.57 [0.36–0.83] | 0.01 |
| NPi at 3h (n=106) | 0.55 [0.34–0.76] | <0.01 | 0.48 [0.23–0.81] | 0.02 |
| NPi at 6h (n=141) | 0.64 [0.48–0.82] | <0.01 | 0.66 [0.45–0.92] | 0.02 |
| NPi at 24h (n=135) | 0.64 [0.47–0.83] | <0.01 | 0.69 [0.46–0.98] | 0.05 |

OR, odds ratio; NPi, Neurological Pupil index.

^a^Multivariable models were adjusted for age, sex, initial GCS score, type of index injury, and time from onset to arrival

Supplemental Table 10. Neurological Pupillary index at each time point and outcome in patients without surgical intervention within 24 hours

| ICU mortality |  |  |  |  |
| --- | --- | --- | --- | --- |
|  | Crude HR | p-value | Adjusted HR* | p-value |
| 0h (n=120) | 0.63 [0.53–0.75] | <0.01 | 0.67 [0.55–0.83] | <0.01 |
| 1h (n=110) | 0.56 [0.46–0.68] | <0.01 | 0.58 [0.46–0.73] | <0.01 |
| 3h (n=104) | 0.48 [0.39–0.69] | <0.01 | 0.50 [0.39–0.64] | <0.01 |
| 6h (n=115) | 0.53 [0.42–0.65] | <0.01 | 0.49 [0.37–0.65] | <0.01 |
| 24h (n=108) | 0.50 [0.39–0.64] | <0.01 | 0.46 [0.34–0.62] | <0.01 |
| Mortality at 6 months |  |  |  |  |
|  | Crude HR | p-value | Adjusted HR* | p-value |
| 0h (n=106) | 0.67 [0.58–0.77] | <0.01 | 0.73 [0.61–0.87] | <0.01 |
| 1h (n=98) | 0.63 [0.54–0.73] | <0.01 | 0.67 [0.55–0.80] | <0.01 |
| 3h (n=91) | 0.53 [0.44–0.62] | <0.01 | 0.53 [0.43–0.65] | <0.01 |
| 6h (n=101) | 0.61 [0.52–0.71] | <0.01 | 0.59 [0.48–0.72] | <0.01 |
| 24h (n=94) | 0.57 [0.48–0.67] | <0.01 | 0.52 [0.41–0.65] | <0.01 |
| Poor functional outcome at 6 months |  |  |  |  |
|  | Crude OR | p-value | Adjusted OR* | p-value |
| 0h (n=106) | 0.63 [0.44–0.85] | <0.01 | 0.61 [0.36–0.95] | 0.04 |
| 1h (n=98) | 0.53 [0.33–0.74] | <0.01 | 0.42 [0.19–0.77] | 0.01 |
| 3h (n=91) | 0.55 [0.34–0.78] | <0.01 | 0.41 [0.17–0.75] | 0.01 |
| 6h (n=101) | 0.57 [0.38–0.77] | <0.01 | 0.45 [0.24–0.71] | <0.01 |
| 24h (n=94) | 0.55 [0.34–0.78] | <0.01 | 0.47 [0.23–0.79] | 0.01 |

HR, hazard ratio; OR, odds ratio; ICU, intensive care unit.

*Multivariable models were adjusted for age, sex, initial GCS score, type of index injury, and time from onset to arrival

Supplemental Table 11. Patient characteristics according to Neurological Pupillary index trajectories

|  | Consistently high (n=87) | Recovered (n=23) | Worsened (n=15) | Consistently low (n=42) | p-value |
| --- | --- | --- | --- | --- | --- |
| Age | 71.3 ± 14.7 | 71.7 ± 11.7 | 67.5 ± 11.6 | 65.0 ± 18.1 | 0.13 |
| Male | 45 (51.7%) | 14 (60.9%) | 7 (46.7%) | 26 (61.9%) | 0.59 |
| Medical history |  |  |  |  |  |
| Hypertension | 50 (57.5%) | 11 (47.8%) | 8 (53.3%) | 22 (52.4%) | 0.85 |
| Diabetes | 36 (41.4%) | 5 (21.7%) | 3 (20.0%) | 17 (40.5%) | 0.16 |
| Stroke | 23 (26.4%) | 5 (21.7%) | 5 (33.3%) | 15 (35.7%) | 0.58 |
| Chronic kidney disease | 9 (10.3%) | 1 (4.35%) | 1 (6.67%) | 9 (21.4%) | 0.19 |
| Malignancy | 13 (14.9%) | 1 (4.35%) | 2 (13.3%) | 7 (16.7%) | 0.57 |
| Smoking | 19 (21.8%) | 7 (30.4%) | 4 (26.7%) | 7 (16.7%) | 0.58 |
| Prior antiplatelet agents | 26 (29.9%) | 6 (26.1%) | 5 (33.3%) | 13 (31.0%) | 0.97 |
| Prior anticoagulants | 8 (9.20%) | 0 (0.00%) | 0 (0.00%) | 6 (14.3%) | 0.16 |
| Premorbid mRS 0-1 | 60 (69.0%) | 16 (69.6%) | 10 (66.7%) | 35 (83.3%) | 0.32 |
| Time from onset to arrival (h) | 2.0 [1.0–6.8] | 1.7 [0.9–6.5] | 1.1 [0.7–2.3] | 1.2 [0.8–4.2] | 0.16 |
| Injury type |  |  |  |  | <0.01 |
| ICH/IVH | 35 (40.2%) | 3 (13.0%) | 7 (46.7%) | 21 (50.0%) |  |
| Ischemic stroke | 22 (25.3%) | 2 (8.70%) | 2 (13.3%) | 2 (4.76%) |  |
| SAH | 9 (10.3%) | 5 (21.7%) | 3 (20.0%) | 3 (7.14%) |  |
| TBI | 21 (24.1%) | 13 (56.5%) | 3 (20.0%) | 16 (38.1%) |  |
| Initial GCS | 9 [7–11] | 8 [6–9.5] | 6 [5–7] | 4 [3–5] | <0.01 |
| Initial GCS, trichotomized |  |  |  |  | <0.01 |
| 3-5 | 12 (13.8%) | 5 (21.7%) | 7 (46.7%) | 33 (78.6%) |  |
| 6-8 | 25 (28.7%) | 8 (34.8%) | 6 (40.0%) | 8 (19.0%) |  |
| 9-15 | 50 (57.5%) | 10 (43.5%) | 2 (13.3%) | 1 (2.4%) |  |
| Surgical treatment within 24h | 26 (29.9%) | 7 (30.4%) | 4 (26.7%) | 10 (23.8%) | 0.91 |

ICU, intensive care unit; mRS, modified Rankin Scale; ICH, intracerebral hemorrhage; IVH, intraventricular hemorrhage; SAH, subarachnoid hemorrhage; TBI, traumatic brain injury; GCS, Glasgow Coma Scale.

Supplemental Table 12. Neurological Pupil index trajectories within 3 hours and outcome

| ICU mortality |  |  |  |  |
| --- | --- | --- | --- | --- |
|  | Crude HR | p-value | Adjusted HR* | p-value |
| Group 1: Consistently high | Reference | - | Reference |  |
| Group 2: Recovered | 0.77 [0.35–1.67] | 0.51 | 0.66 [0.30–1.48] | 0.32 |
| Group 3: Worsened | 4.68 [2.64–8.31] | <0.01 | 5.38 [2.96–9.76] | <0.01 |
| Group 4: Consistently low | 8.18 [5.30–12.62] | <0.01 | 6.78 [4.09–11.25] | <0.01 |
| Mortality at 6 months |  |  |  |  |
|  | Crude HR | p-value | Adjusted HR* | p-value |
| Group 1: Consistently high | Reference | - | Reference |  |
| Group 2: Recovered | 1.20 [0.74–1.93] | 0.46 | 0.91 [0.55–1.52] | 0.73 |
| Group 3: Worsened | 3.07 [1.94–4.85] | <0.01 | 3.91 [2.38–6.40] | <0.01 |
| Group 4: Consistently low | 4.83 [3.48–6.71] | <0.01 | 4.13 [2.79–6.11] | <0.01 |
| Poor functional outcome at 6 months |  |  |  |  |
|  | Crude OR | p-value | Adjusted OR* | p-value |
| Group 1: Consistently high | Reference | - | Reference |  |
| Group 2: Recovered | 1.80 [0.65–5.51] | 0.28 | 2.75 [0.74–11.42] | 0.14 |
| Group 3: Worsened | 8.24 [1.50–154.17] | 0.048 | 11.06 [1.54–233.69] | 0.04 |
| Group 4: Consistently low | 27.90 [5.53–509.29] | <0.01 | 46.65 [5.55–1184.87] | <0.01 |

HR, hazard ratio; OR, odds ratio; ICU, intensive care unit.
*Multivariable models were adjusted for age, sex, initial GCS score, type of index injury, and time from onset to arrival

Supplemental Table 13. Performance metrics of the prediction models

|  | Sensitivity, % (95% CI) | Specificity, % (95% CI) | PPV, % (95% CI) | NPV, % (95% CI) |
| --- | --- | --- | --- | --- |
| ICU mortality |  |  |  |  |
| Model 1: clinical variables only* | 87 (81–93) | 48 (34–62) | 80 (73–87) | 62 (45–77) |
| Model 2: Model 1 + NPi at 0h | 90 (84–95) | 53 (40–67) | 82 (75–88) | 68 (52–83) |
| Model 3: Model 1 + NPi at 24h | 92 (87–97) | 70 (56–85) | 91 (85–96) | 74 (61–88) |
| Model 4: Model 1 + NPi trajectory | 91 (85–96) | 72 (60–84) | 88 (82–94) | 77 (63–88) |
| Mortality at 6 months |  |  |  |  |
| Model 1: clinical variables only* | 68 (57–78) | 65 (53–75) | 69 (59–80) | 63 (51–75) |
| Model 2: Model 1 + NPi at 0h | 79 (69–88) | 68 (57–78) | 74 (65–83) | 73 (61–84) |
| Model 3: Model 1 + NPi at 24h | 87 (80–94) | 59 (46–72) | 75 (66–83) | 77 (65–89) |
| Model 4: Model 1 + NPi trajectory | 84 (75–92) | 67 (55–77) | 74 (65–83) | 78 (67–89) |
| Poor functional outcome |  |  |  |  |
| Model 1: clinical variables only* | 47 (30–63) | 94 (89–98) | 70 (50–88) | 86 (80–91) |
| Model 2: Model 1 + NPi at 0h | 53 (35–69) | 93 (88–97) | 69 (48–86) | 87 (48–86) |
| Model 3: Model 1 + NPi at 24h | 56 (38–72) | 89 (83–95) | 63 (45–80) | 86 (78–92) |
| Model 4: Model 1 + NPi trajectory | 53 (37–69) | 92 (87–97) | 67 (48–84) | 87 (81–93) |

*Clinical variables included age, sex, initial GCS score, type of index injury, and time from symptom onset to hospital arrival
PPV, positive predictive value; NPV, negative predictive value; ICU, Intensive care unit

Supplemental Figure 1. Changes in Neurological Pupil index (NPi) over 24 hours


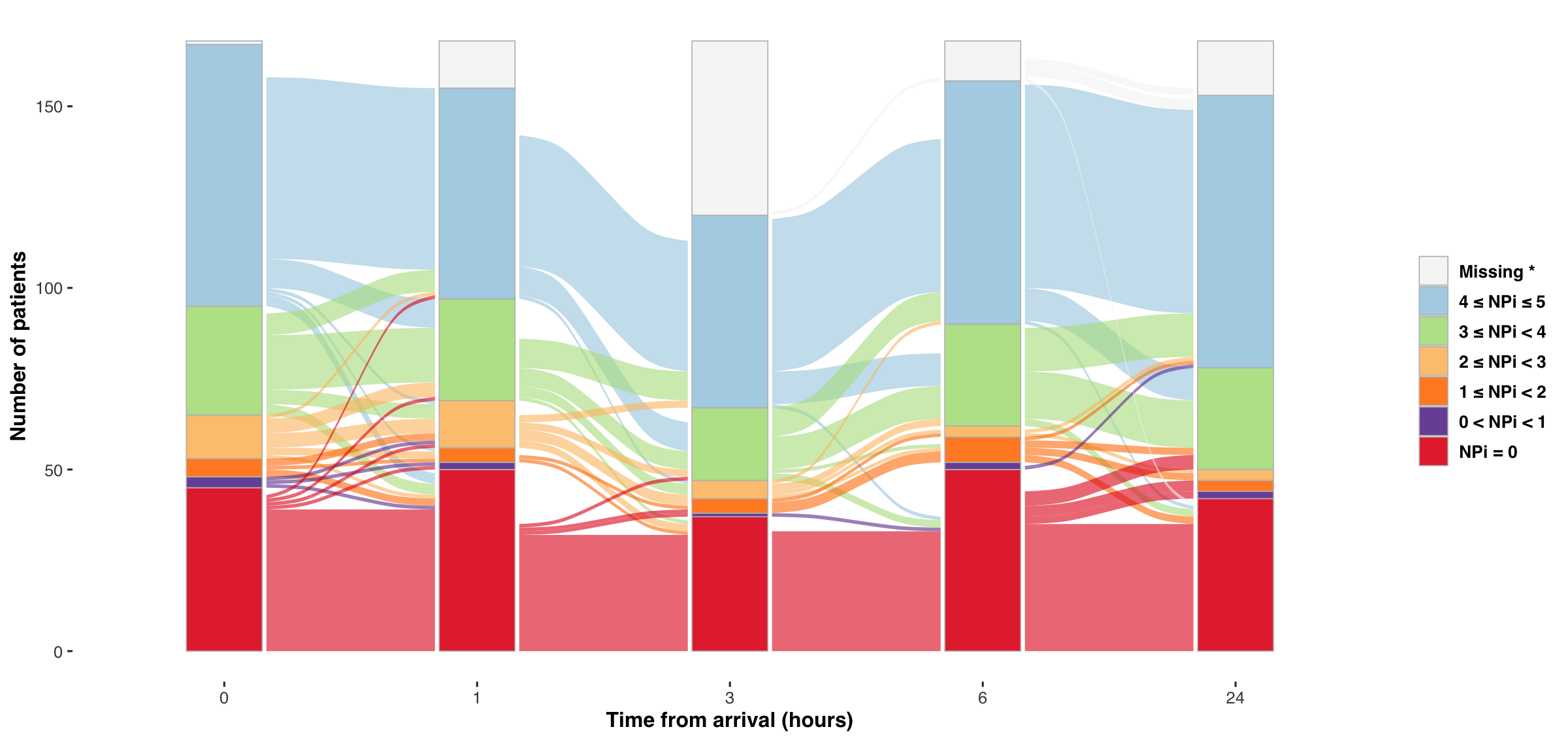


*Missing NPi values were primarily due to transfers to the operating room.

Supplemental Figure 2. Changes in Neurological pupil index (NPi) over time stratified by ICU mortality


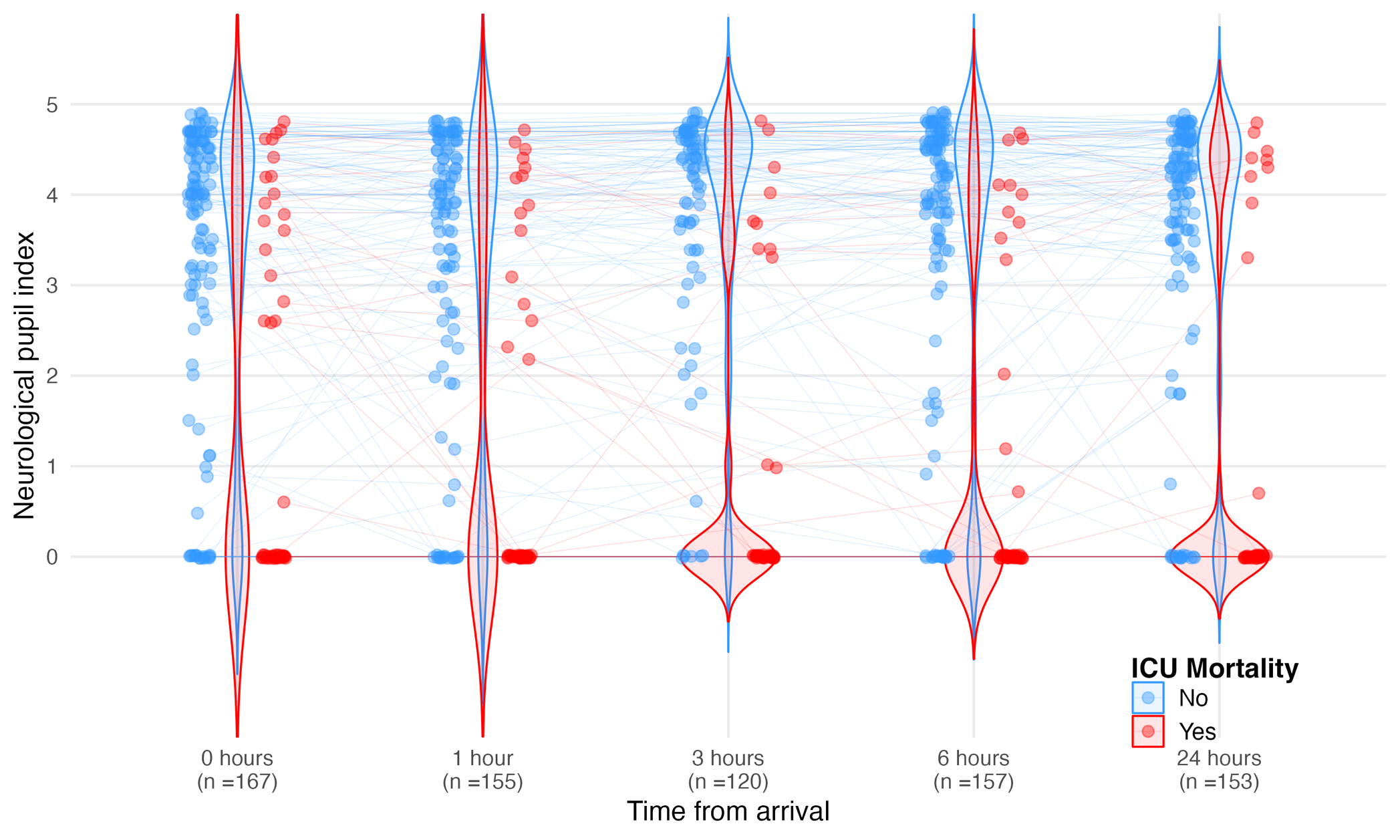


Supplemental Figure 3. Changes in Neurological pupil index (NPi) over time stratified by mortality at 6 months


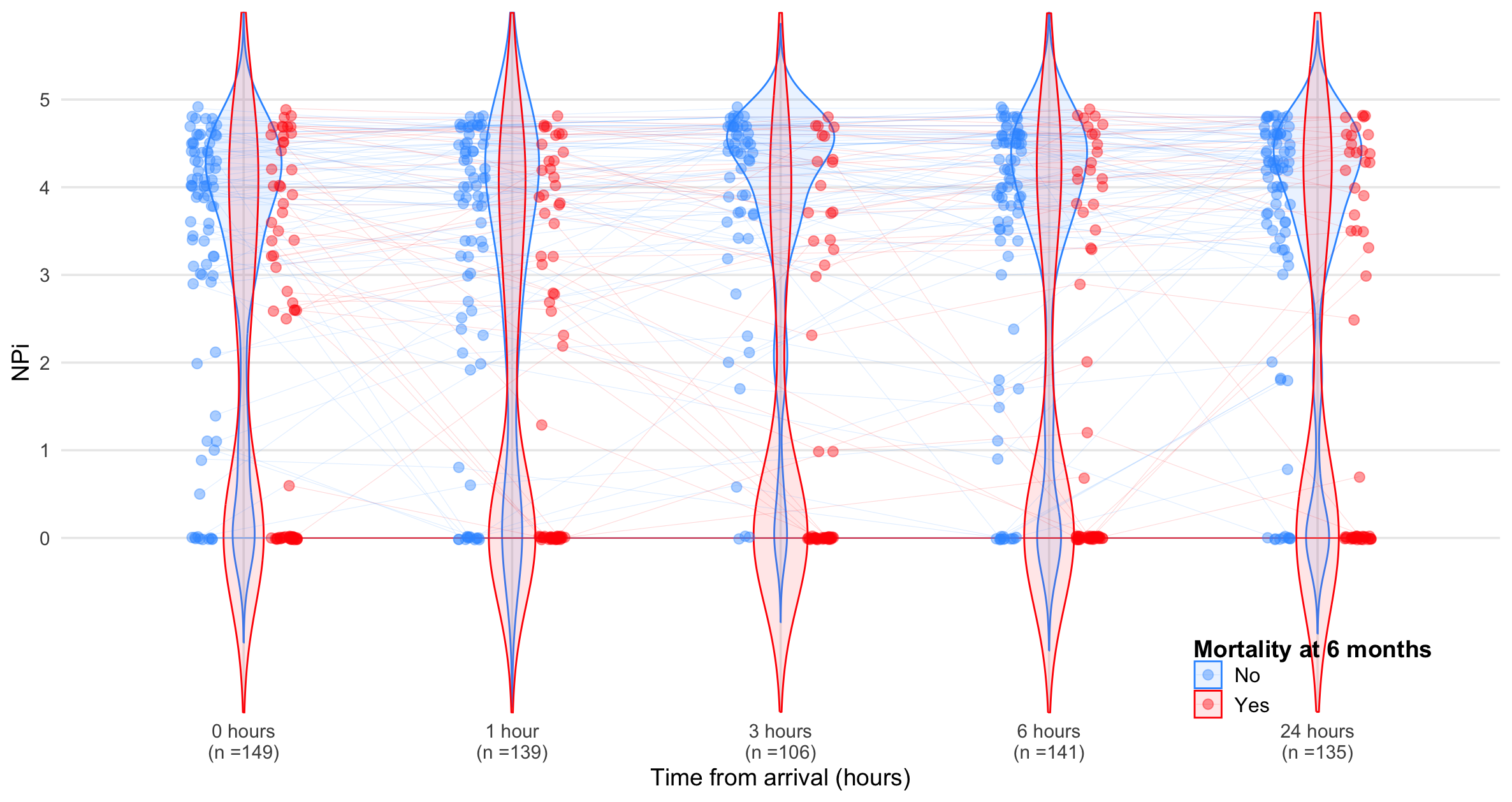


Supplemental Figure 4. Changes in Neurological pupil index (NPi) over time stratified by mRS at 6 months


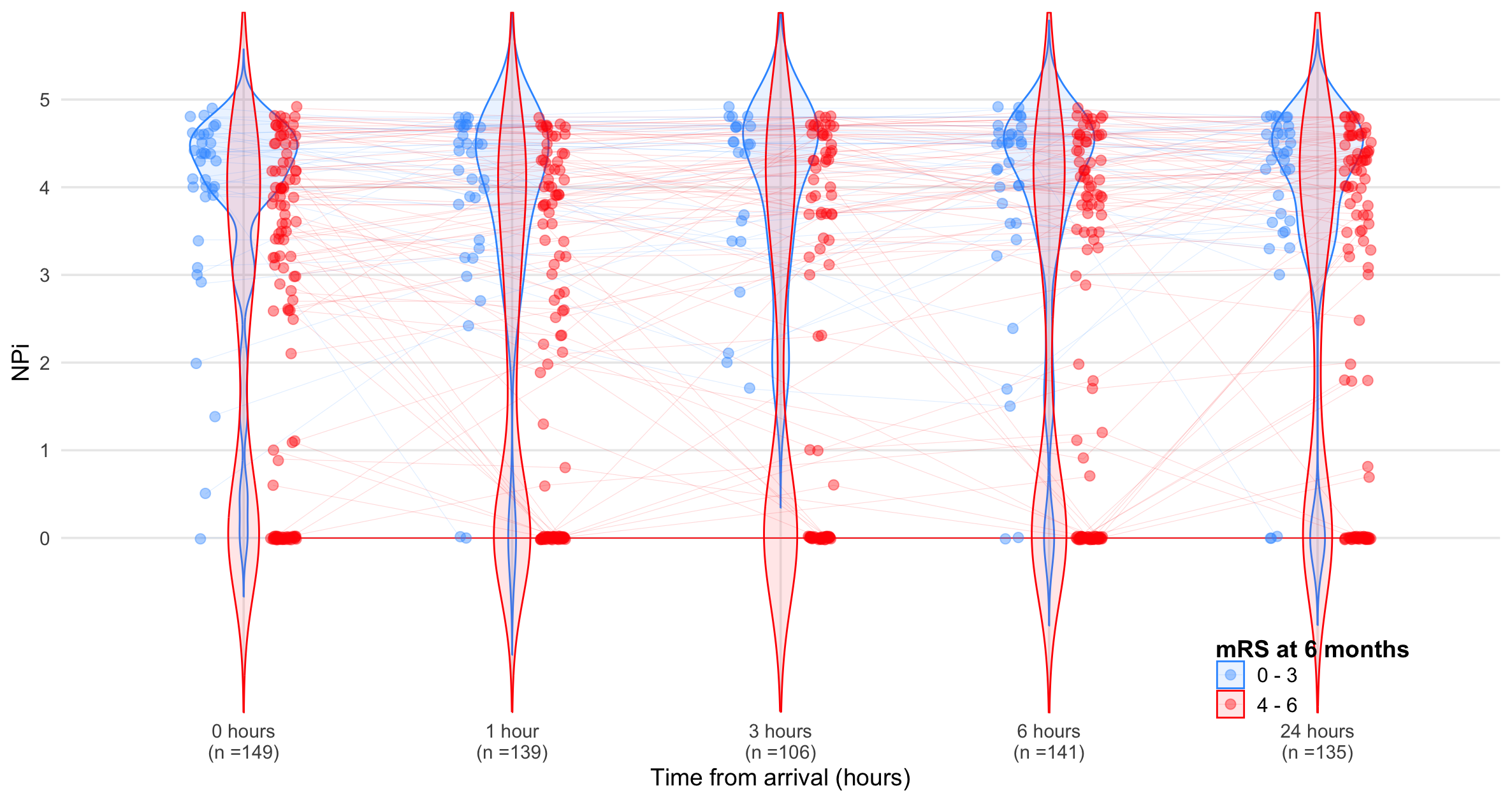

Supplement: Supplementary file 1 [file Supplementary_file_1.docx]
